# A Physiologic Role for Serotonergic Transmission in Adult Rat Taste Buds

**DOI:** 10.1371/journal.pone.0112152

**Published:** 2014-11-11

**Authors:** Luc Jaber, Fang-li Zhao, Tamara Kolli, Scott Herness

**Affiliations:** College of Dentistry, The Ohio State University, Columbus, Ohio, United States of America; Duke University, United States of America

## Abstract

Of the multiple neurotransmitters and neuropeptides expressed in the mammalian taste bud, serotonin remains both the most studied and least understood. Serotonin is expressed in a subset of taste receptor cells that form synapses with afferent nerve fibers (type III cells) and was once thought to be essential to neurotransmission (now understood as purinergic). However, the discovery of the 5-HT_1A_ serotonin receptor in a subset of taste receptor cells paracrine to type III cell suggested a role in cell-to-cell communication during the processing of taste information. Functional data describing this role are lacking. Using anatomical and neurophysiological techniques, this study proposes a modulatory role for serotonin during the processing of taste information. Double labeling immunocytochemical and single cell RT-PCR technique experiments documented that 5-HT_1A_-expressing cells co-expressed markers for type II cells, cells which express T1R or T2R receptors and release ATP. These cells did not co-express type III cells markers. Neurophysiological recordings from the chorda tympani nerve, which innervates anterior taste buds, were performed prior to and during intravenous injection of a 5-HT_1A_ receptor antagonist. These experiments revealed that serotonin facilitates processing of taste information for tastants representing sweet, sour, salty, and bitter taste qualities. On the other hand, injection of ondansetron, a 5-HT_3_ receptor antagonist, was without effect. Collectively, these data support the hypothesis that serotonin is a crucial element in a finely-tuned feedback loop involving the 5-HT_1A_ receptor, ATP, and purinoceptors. It is hypothesized that serotonin facilitates gustatory signals by regulating the release of ATP through ATP-release channels possibly through phosphatidylinositol 4,5-bisphosphate resynthesis. By doing so, 5-HT_1A_ activation prevents desensitization of post-synaptic purinergic receptors expressed on afferent nerve fibers and enhances the afferent signal. Serotonin may thus play a major modulatory role within peripheral taste in shaping the afferent taste signals prior to their transmission across gustatory nerves.

## Introduction

The view of how the taste bud operates has changed dramatically over the last two decades. Once considered a passive recognition unit, the taste bud is now known to be a complicated sensory end-organ composed of elaborate networks of autocrine and paracrine communication pathways that significantly process the gustatory sensory information prior to signaling the central nervous system. These findings have led to the classification of gustatory transduction mechanisms in the taste bud into early and late events [24]. Early transduction events occur between receptor activation by tastant molecules and the resulting depolarization of the taste receptor cell (TRC). Late transduction mechanisms, on the other hand, describe the processing of information among cells of the taste bud by excitatory and inhibitory feedback mechanisms which ultimately shape the neural discharge. A large number of neurotransmitters, neuropeptides, and their corresponding receptors are expressed in defined patterns across the varying cell types of the taste bud, typically referred to as types I, II, and III. Examples include neurotransmitters, such as serotonin, norepinephrine, GABA, and acetylcholine, and neuropeptides, such as cholecystokinin, neuropeptide Y, and vasoactive intestinal peptide. Late transduction events may shape peripheral gustatory signaling through mechanisms that include lateral inhibition, gain modulation, and adaptation. Thus, single TRCs are influenced not only by apical receptors activated by taste stimuli but also through basolateral receptor activation.

Of the multiple neurotransmitters expressed in the taste bud, serotonin ironically remains the best studied yet least understood. Serotonin is expressed in a subset of TRCs (type III cells) which form classic synapses with afferent nerve fibers in a large number of species including mouse, rat, rabbit, and monkey [18], [41], [54], [60], [74], [75]. These cells also express the candidate sour receptor PKD2L1 [30]. Largely because of this classic synaptic morphology, it was long assumed that serotonin was essential to transmission of gustatory information to the central nervous system. ATP is now widely acknowledged as the main gustatory neurotransmitter within the taste bud, acting on P_2_X receptors on afferent nerve fiber terminals [5], [16]. ATP is released from type II cells (cells which express T1R and T2R receptors) in response to tastant stimulation [62]. Release occurs in a calcium-independent but voltage-dependent manner through ATP-release channels. The identity of these channels has been suggested to be connexin or pannexin hemichannels [32], [62] or a newly identified release channel, CALHM1 [72]. Additionally, ATP may participate in cell-to-cell-communication through the activation of P_2_Y and P_2_X receptors expressed on TRCs [7], [15], [20], [31], [38]. Hence, ATP release from type II cells may not only stimulate afferent nerve fibers but additionally stimulate type III cells via cell-to-cell communication by activation of purinergic receptors [3], [38]. This stimulation may then result in serotonin release from these cells [32].

The physiological consequence of this released serotonin remains equivocal. Interestingly, the first demonstration of cell-to-cell communication with the taste bud involved serotonin [25]. At the time, the report that TRCs responded to serotonergic stimulation was unexpected given its putative role as a neurotransmitter to the afferent fiber [19]. In rat posterior TRCs, application of exogenous serotonin results in inhibition of ionic currents that cumulatively would render the cell less responsive to stimuli [25], [26]. These effects were mimicked by 5-HT_1A_ but not by 5-HT_3_ receptor subtype agonists. They were additionally blocked by a 5-HT_1A_ receptor subtype antagonist. Subsequently, this observation has been extended to include a number of species [13], [14], [34], [35] which similarly implicates the 5-HT_1A_ receptor. In rat posterior taste buds, serotonergic TRCs and the 5-HT_1A_ receptor have been demonstrated to be expressed in a paracrine manner [39] suggesting that release of serotonin from type III cells acts to inhibit a subset of neighboring cells. The identity and function of these cells are unknown.

In one scenario, serotonin could be important in transmitting the presence of sour stimuli to ATP-releasing type II cells. Type III cells respond to sour stimuli but do not themselves release ATP; however, blocking purinergic transmission blocks all taste qualities including sour [16]. Therefore, one possibility is that serotonin acts to relay sour information to purinergic type II TRCs which express the 5-HT_1A_ receptor. In another scenario, serotonin may provide a negative feedback pathway onto type II cells that influences ATP release since type III cells additionally express purinergic receptors. In this scenario, serotonin might influence all taste qualities, not just sour. In fact, serotonin is released from TRCs in response to multiple tastant qualities in mouse taste buds [33]. This anatomical arrangement of purinergic and serotonergic crosstalk between type II and type III cells, suggests that serotonin release from type III cells may influence ATP release from type II cells.

The present study explores the hypothesis that serotonin modulates transmission of taste information to the central nervous system by operating during late transduction mechanisms. To investigate serotonin’s role in the peripheral taste system, anatomical, molecular, and physiological techniques were employed to explore the hypothesis that paracrine cell-to-cell communication within the taste bud between the serotonergic TRCs and the 5-HT_1A_ receptor expressing cells plays a physiologically significant role in late transduction mechanisms. Immunocytochemistry and single cell reverse-transcriptase-PCR techniques were employed to phenotype the 5-HT_1A_-expressing cells with respect to co-expression pattern of this receptor relative to distinct phenotypic markers associated with specific cells types and with the transduction of specific tastes. Additionally, whole nerve neurophysiological recordings were employed to study the effect of blocking serotonergic transmission on gustatory afferent nerve responses. We conclude that serotonin facilitates gustatory responses during late transduction mechanisms by exerting an inhibitory action on type II cells of the bud which serves to regulate ATP release from these cells and thus prevents desensitization of purinergic receptors on the afferent nerve fiber.

## Materials and Methods

Serotoninergic signaling mechanisms in the rat taste bud were studied with a combination of histological, molecular, and physiological approaches. Adult male Sprague–Dawley rats served as subjects for all experiments. All procedures were approved by The Ohio State University's Institutional Animal Care and Use Committee (Protocol ID 2009A0026-R1).

### Immunocytochemistry

The expression pattern of the 5-HT_1A_ receptor throughout the oral cavity and its co-localization with TRC phenotypic markers were studied using single and double labeled immunofluorescent techniques. Subjects were sacrificed by decapitation after first achieving surgical level of anesthesia with a Ketamine (91 mg/ml)/Acepromazine (0.09 mg/ml) mixture (0.09 ml/100 gm BW). Harvested tissue included anterior tongue, nasoincisor duct, palate, foliate, and circumvallate papillae. For experiments using anti-serotonin antibodies, animals were preloaded with a 5-HT precursor 5-hydrxytryptophan (80 mg/kg, *i.p.* injection 1 hr prior to sacrifice). Tissue was immersion fixed in Bouin’s fixative (5 hrs, 4°C), paraffin embedded, and sectioned at 4 micron thickness. Conventional and TSA amplified immunocytochemistry (ICC) were conducted as previously described using fluorescent microscopy [39]. For double labeling immunocytochemistry with primary antibodies raised in different species, sections were blocked in 10% normal serum of species matching the secondary fluorescent antibody (1 hr, RT). Sections were then incubated in primary antiserum (150 µL per slide, diluted in PBS containing 2% normal serum) at the appropriate dilutions. Antibody dilutions were empirically adjusted to minimize cross reactivity and background staining. Descriptions of primary antibodies are presented in Table 1. The following day, sections were rinsed in PBS and incubated with Cy3- or FITC-conjugated secondary antibody (150 µL per slide, 1∶400 in PBS containing 1.5% normal serum, 1 hr, RT) in the dark. Slides were then washed in PBS (3×10 min) and incubated in the second primary antiserum at the appropriate dilution (24 hr, 4°C). The following day, slides were washed in PBS and incubated with the second secondary antibody (1∶400, 1 hr) in the dark, washed in PBS, mounted in Fluoro-Gel, and observed under a fluorescent microscope equipped with a digital camera (Nikon EFD-3, Japan).

**Table 1:** List of primary antibodies used in the present study.

Antigen	Immunogen	Manufacturer, species, type,catalog number	Dilution
*G_α gust_*	A peptide mapping within a highly divergentdomain of G_α gust_ of rat origin	Santa Cruz Biotech, rabbit polyclonal, affinity purified IgG, sc–395	1∶1000
*SNAP-25*	Human crude synaptic immunoprecipitate thatrecognizes SNAP-25 Protein	Millipore, mouse monoclonal, MAB331	1∶300
*NCAM*	Highly purified chicken NCAM	Chemicon, rabbit polyclonal, immunoaffinity purified, AB5032	1∶300
*NPY*	Rabbit NPY coupled to methylatedBSA with glutaraldehyde	Immunostar, rabbit polyclonal, affinity purified, 22940	1∶300
*NPY1R*	Recognizes amino acids 356–382of the rat NPY1 receptor	Immunostar, rabbit polyclonal, affinity purified, 24506	1∶300
*CCKAr*	Synthetic peptide rat CCK-A R. Detects CCK-Areceptor amino acids 256–267 in rat	Neuromics, affinity purified, rabbit polyclonal	1∶300
*CCK-8*	Recognizes octapeptide CCK8	Chemicon, rabbit polyclonal, AB1973	1∶300
*5HT*	Serotonin conjugated to BSA. Recognizesserotonergic sites is fixed tissue sections	Millipore, rat monoclonal, MAB352	1∶150
*5HT_1A_*	Synthetic peptide corresponding to a region locatedin the large third intracellular loop of the rat andmouse 5-HT_1A_ receptor protein	Chemicon, rabbit polyclonal, AB15350	1∶150

When it was not possible to acquire two primary antibodies raised in different species, an indirect immunofluorescence double-labeling protocol was employed which allowed detection of two antigens with primary antibodies raised in the same species by altering the dilution and detection method for each antigen with either conventional or Tyramide Signal Amplification (TSA Biotin System, PerkinElmer, Waltham MA, USA). One primary antibody is used at very high dilution with TSA detection whereas the second primary is used with standard dilution and detection methods. Streptavidin-conjugated IgG Fab fragment (instead of a fluorophore-conjugated one) is used to detect the first primary antibody so that the second primary antibody can be used at very high dilution thus minimizing interference or cross-reaction between the first primary antibody and the second secondary antibody [66]. This method also prevents the first secondary antibody from reacting with the second primary antibody.

Rehydrated deparaffinized tissue sections were treated with a 0.5% hydrogen peroxide in methanol (30 min) to eliminate endogenous peroxidase activity. Sections were subsequently incubated in PBS containing 10% normal serum (1 hr, RT) to reduce nonspecific antibody binding. Primary antiserum (diluted in PBS containing 2% normal serum) was applied and slides were housed in a closed moist chamber (36 hr, 4°C). After PBS washing, sections were incubated with biotin-streptavidin-conjugated IgG Fab fragment (1∶800 in PBS containing 1.5% normal serum, 1 hr, RT) and processed according to the kit’s instructions (NEN Life Science Products, Boston, MA, USA). Slides were mounted in Fluoro-Gel and observed under the fluorescent microscope.

Control slides were included in every experiment to ensure that fluorescent signals did not arise from cross-reactivity of secondary antibodies with the inappropriate primary antibody. To control for false positives and non-specific binding in single-label ICC experiments, two slides were included in each experiment; the first slide omitted the primary antibody and the second slide omitted the secondary antibody. Similarly, for double-label ICC experiments (including TSA experiments), four slides were included; the first slide lacked the first primary, the second lacked the second primary, the third lacked the second primary, and the fourth slide lacked the second secondary antibody. In all control slides, cells were expected to lack positive staining. As a criterion for acceptable labeling, the emitted signal of the secondary antibody was expected to be visible only in the emission spectrum matching its fluorophore. If color bleeding to the emission spectrum of the alternate secondary antibody was visible, adjustments were made in the dilutions of the primary antibody.

Data were analyzed by counting individual taste buds and TRCs. To avoid counting the same cell more than once, only every fourth consecutive section on each slide was chosen for data analysis. This ensured that the sections being considered for cell counting were separated by at least 12 µm, greater than the diameter of a taste cell. Taste buds were analyzed twice to confirm the cell count. Only cells that showed clear apical and/or perinuclear labeling were considered immunopositive. All cell counts were performed under 40X magnification. A limitation for direct microscopic cell counting was that it only accounted for stained cells within the visible plane of the 4 µm-thick section and it did not fairly represent all labeled cells within the actual tri-dimensional frame of the taste bud. Antigen-expressing cells cannot be presumed to be uniformly distributed throughout the taste bud. Consequently, consecutive tissue sections would most likely show discrepancies in the number of labeled cells. This method therefore serves as an approximate representation of the actual number of immunopositive cells. Digital photos of immunofluorescent cells were captured under the 40X objective lens by a digital camera (Photometrics CoolSNAP. Tucson, AZ), processed using MetaMorph software (Molecular Devices LLC, Sunnyvale, CA, USA), and subsequently imported into imaging software (Canvas Illustrator; ACD systems, USA).

### Single Cell RT-PCR

Individual TRCs were phenotyped using single cell RT-PCR reactions. RNA was extracted from single TRCs dissociated from circumvallate papillae, used as template in a two-round cDNA synthesis process, followed by standard PCR reactions with primers for specific cell markers. Single dissociated TRCs were collected under microscopic examination from a total of ten animals. Each TRC was carefully suctioned into a glass pipette (∼8 µm-diameter) in less than 20 nanoliters of ECF solution. The pipette tip was examined at 100X magnification using an inverted bifocal microscope to verify the presence of a single TRC. The majority of harvested TRCs had a morphological appearance consistent with type II cells, as indicated by a circular soma and short cellular processes. The pipette solution containing the single cell was expelled into a PCR tube containing 3 µl of RNA extraction solution. The cell was lysed by vigorous vortex mixing (1 minute RT).

RNA was first amplified in a linear fashion prior to cDNA synthesis using the MessageBOOSTER cDNA Synthesis from Cell Lysates Kit (EPICENTRE Biotechnologies, Madison, WI, USA). Prior to cDNA synthesis, template RNA was desalted and purified from potential genomic DNA contamination using RNase-free DNase. First-strand cDNA was reverse-transcribed from template RNA by adding T7-oligo(dT) primer, annealing at 65°C for 5 min and then incubating the reaction with MMLV reverse transcriptase at 37°C for 60 min. Second-strand cDNA synthesis was then performed by adding DNA polymerase and incubating at 65°C for 10 min. The reaction was terminated by heating at 80°C for 3 min. cRNA was then transcribed from cDNA by incubating the reaction with RNA polymerase at 42°C for 4 hrs. RNase-free DNase was subsequently added to remove any remaining DNA (37°C for 15 min). The transcribed RNA was purified and desalted using the RNeasy MinElute Cleanup Kit (Qiagen, Inc., Valencia, CA, USA). RNA was washed and centrifuged in RNA spin columns with a succession of buffers and ethanol solutions (RLT, RPE, 100° and finally 80° ethanol), eluted in a final volume of 14 µL nuclease-free H_2_O, concentrated in an Eppendorf Vacufuge (3 to 8 µL final volume), and then used in the second round of 1st-strand cDNA synthesis with random primers and MMLV reverse-transcriptase (37°C, 1 hr). Finally, RNase H was incubated with the reaction for 20 min at 37°C to eliminate any remaining RNA and the reaction was terminated at 95°C for 2 min.

The expression of specific phenotypic markers in a single TRC was investigated using custom-designed forward and reverse primers. Primer sequences are described in Table 2. Primer sets included 5-HT_1A_ and taste transduction receptor molecules T1R3, T2R9, α-gustducin, PKD2L1, and GAD. Three sets of 5-HT_1A_ primers corresponding to three different sequences within the 5-HT_1A_ gene were tested in whole taste papilla and single cell RT-PCR (termed 5-HT_1A_[i], 5-HT_1A_[ii], and 5-HT_1A_[iii]). These primers were tested in a group of 10 single cells extracted from the CV papillae of five animals (group “A”), along with primers for α-gustducin, T1R3, and T2R9.

**Table 2:** Primer sequences used in RT-PCR reactions.

Target	Accession number	Primers	Product size	Reference
*T1R3*	NM_130818	CCTCTTCTGCCTCAGTGTCC TAAGCTAGCATGGCGAAGGT	468 bp	[10]
*T2R9*	NM_023999	TTTCATGGGCAATCTCCTTC CATGTGGCCCTGAGATCTTT	514 bp	[78]
*CK8*	NM_199370	ATGCAGAACATGAGCATC ACAGCCACTGAGGCTTTA	440 bp	[42]
*Gustducin*	X65747	GTTGGCTGAAATAATTAAACG ATCTCTGGCCACCTACATC	251 bp	[50]
*5HT_1A_(A)*	NM_012585.1	CAGAGGAAGGTGCTCTTTGG AAGAAGAGCCTGAACGGACA	171 bp	[76]
*5HT_1A_(B)*	NM_012585.1	CAGAGGAAGGTGCTCTTTGG AGCTTAGGAACTTCGTCGGCA	201 bp	[76]
*5HT_1A_(C)*	NM_012585.1	GGCAGCCAGCAGAGGATGAA CCCCCCAAGAAGAGCCTGAA	336 bp	[6]
*PKD2L1*	NM_001106352.1	GAGCTGGTCTTCTTTGTCCG CTGCAGTCTCCTTCCAGACC	266 bp	[17]
*GAD*	NM_012563.1	TCTTTTCTCCTGGTGGTGCC CCCCAAGCAGCATCCACAT	373 bp	[37]

The standard PCR reaction mixture contained 10 µL iQ SYBR Green Supermix (Bio-Rad Laboratories, Hercules, CA, USA), 1 µL of template cDNA, 1 µL each of 100 µM forward and reverse primers, and 7 µL nuclease-free water. Template cDNA that was produced from total bud RNA was diluted at 1∶5 for RT-PCR; cDNA produced from a single TRC RNA was used undiluted. The PCR profile was 3 min at 95°C (initialization step, one cycle), 15 sec at 95°C (denaturation step, 40 cycles), 30 sec at 58°C (annealing step, 40 cycles), 30 sec at 72°C (extension step, 40 cycles), and 30 sec at 72°C (final elongation step, 1 cycle). PCR products were separated by gel electrophoresis on a 1.5% agarose gel containing 0.5 µg/ml ethidium bromide. Each lane was loaded with a mixture containing 5 µl of PCR product, 4 µl double-distilled H_2_O and 1 µl gel loading buffer (Invitrogen 10X Blue Juice, Life Technologies, NY, USA). Bands were measured relative to a 100 bp DNA ladder (Lonza SimplyLoad DNA ladders, Rockland, ME, USA).

PCR reactions were optimized on cDNA synthesized from RNA extracted from whole posterior papillae. In addition to performing whole taste papilla RT-PCR experiments as positive control, primers for cytokeratin 8 (CK8), a protein ubiquitously expressed across all mature TRCs, were included in every single-cell RT-PCR run as a positive control. Negative control reactions, to ensure that the amplified product did not result from genomic or extraneous DNA, included reactions lacking primers or DNA template. The latter contained a small aliquot of ECF (in lieu of the DNA template) sampled adjacent to the collected cell. Single cells were only included in the data set if they satisfied criteria for both positive and negative controls.

### Chorda tympani neural recordings

Chorda tympani neurophysiological recordings were performed as previously described [23]. Subjects, weighing between 160 and 300 gm, were anesthetized with 50 mg/kg pentobarbital (Nembutal 50 mg/ml, Ludbeck Inc, USA) and supplemented with 0.05 ml every thirty minutes or as needed to maintain surgical level of anesthesia. The animal was secured with a non-traumatic head holder and tracheotomized. The left external jugular vein was catheterized with 15 cm polyethylene tubing (2 mm internal diameter) filled with heparinized saline solution. Body temperature was maintained at 36–37°C. Animals were sacrificed at the end of the recording session by decapitation while under surgical level of anesthesia.

The chorda tympani was exposed from the point where it leaves the tympanic bulla caudally to the point where it separates from the lingual nerve rostrally. It was freed of surrounding fascia and connective tissue and severed at the caudal end. The peripheral end of the nerve was carefully desheathed of its epineurium. Neural recordings were made between two platinum iridium electrodes using an AC preamplifier (A-M systems Inc. Model 3000) at a gain of 500X and filtered between low pass and high pass bandwidths of 3 kHz and 100 Hz respectively. Signals were full-wave rectified and integrated with a time constant of 0.2 sec on a wide band AC to DC converter (GRASS systems, model 7P3B) and displayed on an ink recording chart.

Stimuli were presented to the anterior tongue via a Pasteur pipette. Gustatory stimuli (0.5 M NaCl, 1.0 M sucrose, 0.03 M quinine HCl, or 0.01 N HCl) were prepared with reagent grade chemicals in distilled water. The chosen concentrations of taste solutions produced greater than half-maximal integrated whole nerve responses based on results from previous CT recording studies [4], [65], [67]. Stimuli were delivered in the following sequence: NaCl, sucrose, quinine, and then HCl, with a two-minute interval between consecutive stimuli.

Stability criteria of neural responses had to be demonstrated prior to drug delivery. These criteria included a steady baseline between stimuli, consistent response magnitude to three sets of taste stimuli delivery, and overall consistency in the shape of the neural responses. Once these criteria were satisfied, experimental drug or control vehicle was administered into the jugular vein in the form of a single 300 µL bolus injection delivered over a period of 5 seconds. Nerve responses to taste stimuli were monitored and recorded in the periods before, and at least up to one hour following the injection to examine the immediate, as well as the long term effects of the injected solution.

The tonic portion of the integrated neural response was selected for data analysis. The amplitude was measured in arbitrary units between baseline and the midpoint on a line drawn through the response plateau. Magnitudes of the three responses taken just before drug injection were averaged and their mean was compared with that of the averaged amplitude of the first three responses for that stimulus taken immediately after the drug injection. Post-injection data were normalized to the pre-injection mean, thus the normalized value of post-injection that had a value of 1.0 indicated no change in mean-response. A mean value above 1.0 signified an increase in post-injection response magnitude and a mean below 1.0 signified a decrease. Relative data were plotted as response means ± standard errors.

Statistical analysis was performed with Shapiro-Wilk’s normality test with the following null hypothesis (Ho): the population has a normal distribution. The test resulted in *p* values that were always greater than 0.05, which implied that the alternative hypothesis should be rejected. Since the goal was to compare the means of two variables and test if the average difference is significantly different from zero, the paired T-test was chosen for statistical analysis with the following null hypothesis (Ho): mean response-amplitudes corresponding to two different stages of recording were not significantly different. A two-tailed significance (2-tailed *p* value) that was ≥0.05 signified that the null hypothesis is valid and that the difference between the two means being compared was not statistically significant.

## Results

### Phenotypic expression patterns of the 5-HT_1A_ receptor in taste buds

Cellular and molecular experiments were performed to explore the expression pattern of the 5-HT_1A_ receptor in taste buds across anterior and posterior gustatory fields and to examine the co-expression patterns of 5-HT_1A_–expressing TRCs with known TRC-type phenotypic markers and taste receptor molecules.

#### 5-HT1A expression throughout the oral cavity

**Figure 1:**
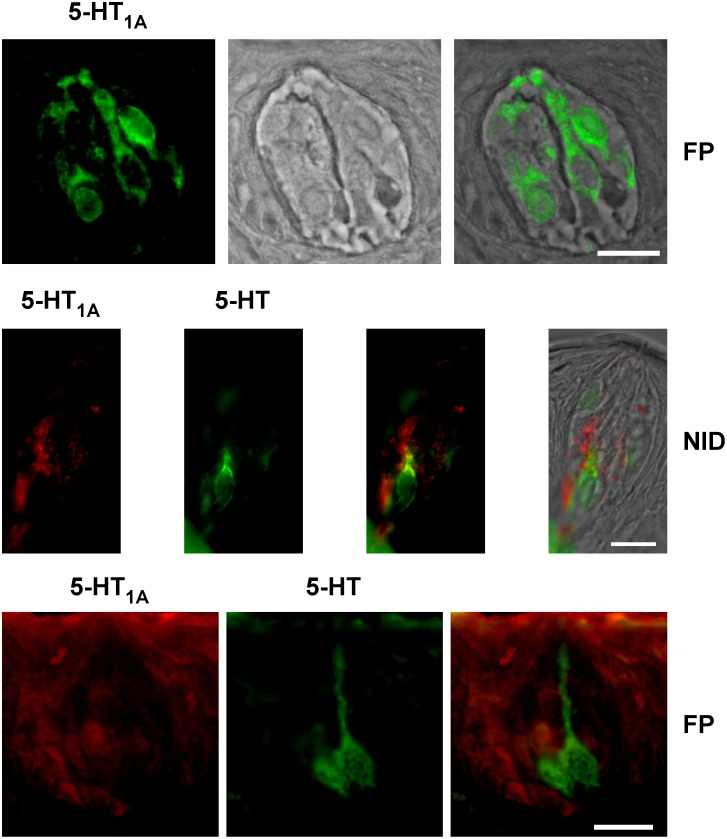
To confirm expression of the 5-HT_1A_ receptor in anterior gustatory fields, taste buds of the fungiform papillae (FP), nasoincisor ducts (NID), and soft palate were examined using immunocytochemistry. Illustrated is single labeling ICC overlaid on a bright field image for a single taste bud from the anterior tongue (top). In double labeling experiments, paracrine expression of 5-HT and 5-HT_1A_ was observed in taste buds of both the fungiform papillae and in the NID. Expression of the 5-HT_1A_ receptor was not observed in taste buds of the palate (not illustrated). Scale bars are 15 microns.

While previous work in posterior taste buds has demonstrated the expression of 5-HT and the 5-HT_1A_ receptor in a paracrine manner, no data exist on 5-HT_1A_ expression in anterior gustatory fields. To investigate these fields, taste buds from the fungiform papillae (FP), nasoincisor duct (NID), and soft palate were examined using a single-label immunocytochemical approach with either a 5-HT or a 5-HT_1A_ antibody. Clearly labeled 5-HT or 5-HT_1A_ immunopositive cells were observed using immunofluorescence in taste buds of FP and the NID (Figure 1). In all cases, 5-HT-immunopositive TRCs featured tall and slender cell bodies and relatively small nuclei, characteristics of type III cells. On the other hand 5-HT_1A_-immunopositive TRCs were relatively shorter and with well-formed roundly-shaped soma, as commonly observed in type II cells. In fungiform papillae, an average of about two 5-HT-immunopositive TRCs (1.83±0.03; n = 3 animals) and about two 5-HT_1A_-immunopositive TRCs (1.97±0.05; n = 3) were detected per cross-sectioned taste bud. In taste buds of the NID, an average of one (1.1±0.1; n = 3) 5-HT_1A_-immunopositive cell was detected per cross-sectioned taste bud. 5-HT_1A_ immunopositive cells were not observed in taste buds of the soft palate (n = 3). To examine the co-expression pattern of 5-HT and 5-HT_1A_ in the anterior field, double label immunocytochemistry experiments were performed on fungiform and NID taste buds. In all instances of these experiments, immunopositive TRCs to each antibody were observed in non-overlapping subpopulations of TRCs (Figure 1) in taste buds of fungiform papillae and the nasoincisor duct. These data suggest that the 5-HT_1A_ receptor is distributed in the anterior field in a manner similar to foliate and circumvallate taste buds. This receptor may not be expressed in the soft palate.

#### 5-HT1A expression and cell type

To investigate the cell type expression of the 5-HT_1A_ receptor, double label immunocytochemistry experiments were conducted on taste buds from anterior and posterior gustatory fields with the phenotypic cell type markers gustducin, a marker of a subset of type II cells, or NCAM, a marker of type III cells in rat.

**Figure 2:**
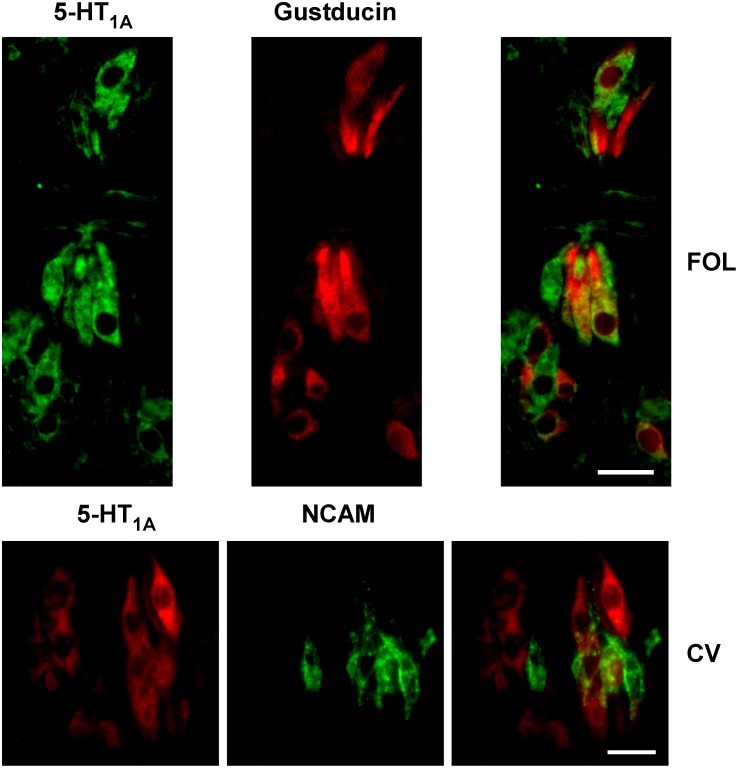
To examine the expression of the 5-HT_1A_ receptor across taste receptor cell types, double label immunocytochemistry experiments were performed with alpha-gustducin, a type II cell marker, and NCAM, a type III cell marker. 5-HT_1A_-expression extensively overlapped with expression of alpha-gustducin. Illustrated (top) is an example from foliate papillae (FOL). On the other hand, no overlapping expression was observed for 5-HT_1A_ and NCAM, illustrated at bottom with tissue from the circumvallate papillae (CV). Scale bars are 15 microns.

A majority of 5-HT_1A_-immunopositive TRCs co-expressed gustducin in anterior and posterior fields. These immunopositive cells displayed emblematic morphology of type II cells (Figure 2). In anterior fields, about 82% of all 5-HT_1A_ cells co-expressed gustducin (n = 44 cells from 56 cross sectioned taste buds) whereas in posterior fields, about 70% of 5-HT_1A_ cells co-expressed gustducin (n = 91 cells from 92 taste buds). Data are summarized in Table 3. On the other hand, fewer gustducin-positive cells co-expressed 5-HT_1A_ in anterior fields whereas comparable numbers were expressed in posterior fields. About 37% of gustducin cells co-expressed 5-HT_1A_ in anterior taste buds and 75% in posterior taste buds. 5-HT_1A_ immunopositive cells were not observed to co-express NCAM. In 180 cross sectioned taste buds from posterior field, 135 5-HT_1A_-immunopositive cells and 175 NCAM immunopositive cells were observed. None displayed overlapping expression. Since reliable NCAM immunostaining could not be produced in anterior tongue tissue, data were only obtained from posterior tongue tissue. Taken together, these results confirm 5-HT_1A_ expression in type II cells, agreeing with our previous suggestion [39], and exclude its expression in type III cells.

**Table 3:** Immunocytochemical cell counts of co-expression patterns of 5HT_1A_ expressing taste receptor cells.

		Total tastebuds	5-HT_1A_labeled cells	PhenotypicMarker	Double-labeled cells	Mean 1 (%DL/5-HT_1A_/TBs/section) ± SE	Mean 2 (%DL/p.m/TBs/section) ± SE
***Anterior tongue:*** ***fungiform papillae +*** ***NID (n = 8 animals)***	5-HT_1A_/NPY	120	130	190	95	73.08±2.90	50.00±9.50
	5-HT_1A_/NPY	60	60	48	36	60.00±2.19	75.00±0.98
	5-HT_1A_/NPY1R	48	96	80	56	58.33±3.70	70.00±2.03
	5-HT_1A_/GUS	56	44	96	36	81.82±0.63	37.50±6.93
	5-HT_1A_/CCK-8	54	48	42	36	75.00±0.98	85.71±0.46
***Posterior tongue:*** ***CV & foliate papillae*** ***(n = 8 animals)***	5-HT_1A_/NPY	60	70	142	59	84.29±0.85	41.55±9.11
	5-HT_1A_/NPY1R	84	59	80	46	77.97±1.04	57.50±3.17
	5-HT_1A_/NCAM	180	135	175	0	0.00	0.00
	5-HT_1A_/SNAP	142	150	162	134	89.33±1.20	82.72±2.18
	5-HT_1A_/GUS	92	91	84	63	69.23±2.38	75.00±1.71
	5-HT_1A_/CCK-8	84	81	138	57	70.37±0.00	41.30±0.00

**Figure 3:**
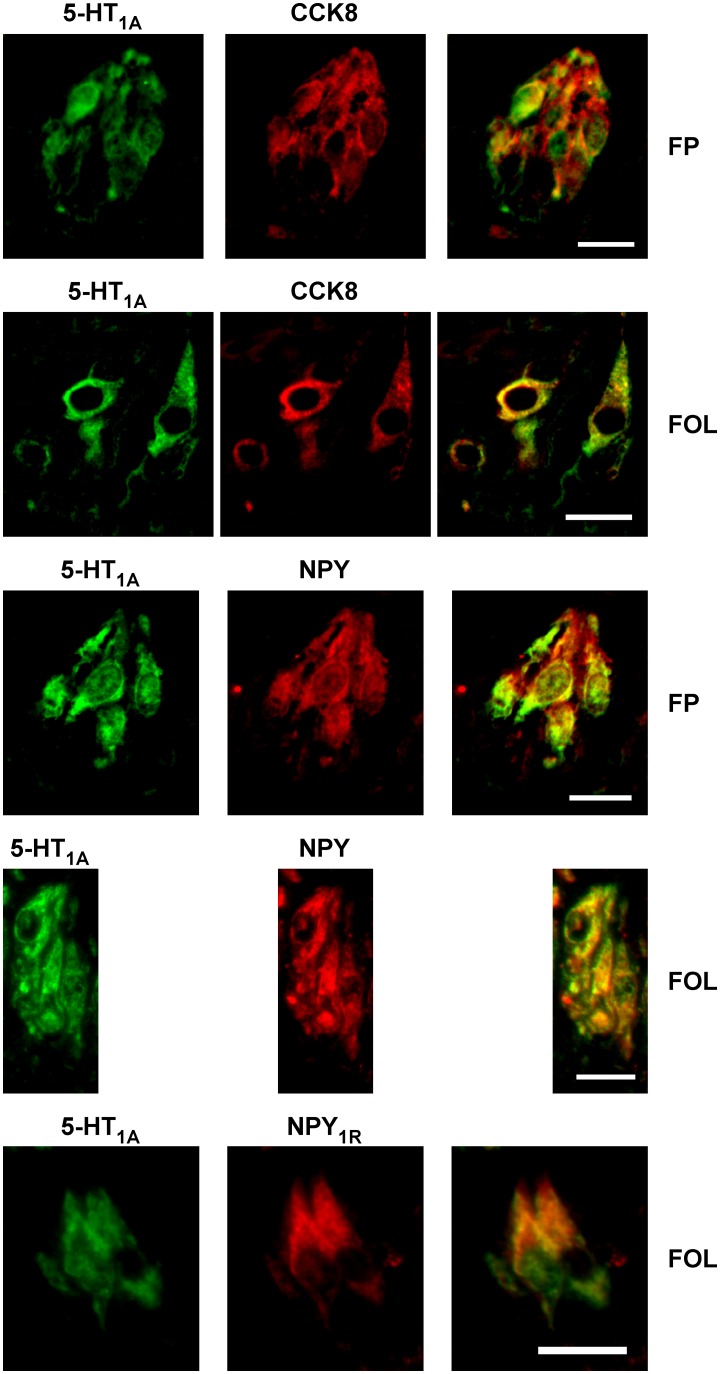
5-HT_1A_ expression displayed extensive overlapping expression with neuropeptides cholecystokinin-8 (CCK8), neuropeptide Y (NPY) and the neuropeptide Y-1 receptor (NPY_1R_). Immunocytochemical double labeling examples are presented for 5-HT_1A_ and CCK8 in fungiform (FP) and foliate (FOL) papillae, for 5-HT_1A_ and NPY in fungiform and foliate papillae, and for 5-HT_1A_ and NPY-1R in foliate papillae. Since these neuropeptides are known to be expressed in type II cells, these co-expression patterns corroborate expression of 5-HT_1A_ in type II cells. Scale bars are 15 microns.

Since type II cells are heterogeneous, additional rat type II phenotypic markers were tested [24]. These included cholecystokinin 8 (CCK-8), neuropeptide Y (NPY), and the NPY-1R receptor. 5-HT_1A_ expressing cells in fungiform taste buds significantly co-expressed CCK-8 (Figure 3, Table 3). Seventy-five percent of 5-HT_1A_ positive cells co-expressed CCK-8 in anterior taste buds (36 of 42 immunopositive TRCs) and 70% of 5-HT_1A_ positive cells co-expressed CCK-8 in posterior cells (57 of 81 immunopositive TRCs). Not all CCK-8 positive cells expressed 5-HT_1A_. Most anterior CCK-8 cells co-expressed 5-HT_1A_ (85%; 36 of 42 cells) whereas fewer posterior CCK-8 cells co-expressed 5-HT_1A_ (41%; 57 of 138 TRCs). A similar expression pattern was noted for NPY. NPY-expressing TRCs are a subset of CCK cells (i.e., all NPY cells co-express CCK) so strong overlapping expression with 5-HT_1A_ was expected. In anterior fields, 60% of 5-HT_1A_ cells co-expressed NPY (36 of 60 TRCs) whereas 84% (59 of 70 TRCs) did so in the posterior field. Similar to CCK results, most NPY cells in anterior fields co-expressed 5-HT_1A_ (75%; 36 of 48 TRCs) whereas fewer did so in posterior taste buds (41%; 59 of 142 TRCs). NPY-1R, which is expressed paracrine to NPY cells, similarly had strong overlapping expression with 5-HT_1A_. In the anterior field, about half of the 5-HT_1A_ cells expressed NPY-1R (58%; 56 of 96 TRCs) with greater co-expression observed in posterior taste buds (85%; 59 of 70 TRCs). Conversely, in anterior TRCs, 75% of NPY-1R TRCs co-expressed 5-HT_1A_ (36 of 48 TRCs) compared to 42% (59 of 142 TRCs) in posterior taste buds. Thus, peptide co-expression patterns with 5-HT_1A_ confirmed previous gustducin results and were highly consistent the known co-expression patterns with CCK, NPY, and NPY-1R among themselves.

**Figure 4:**
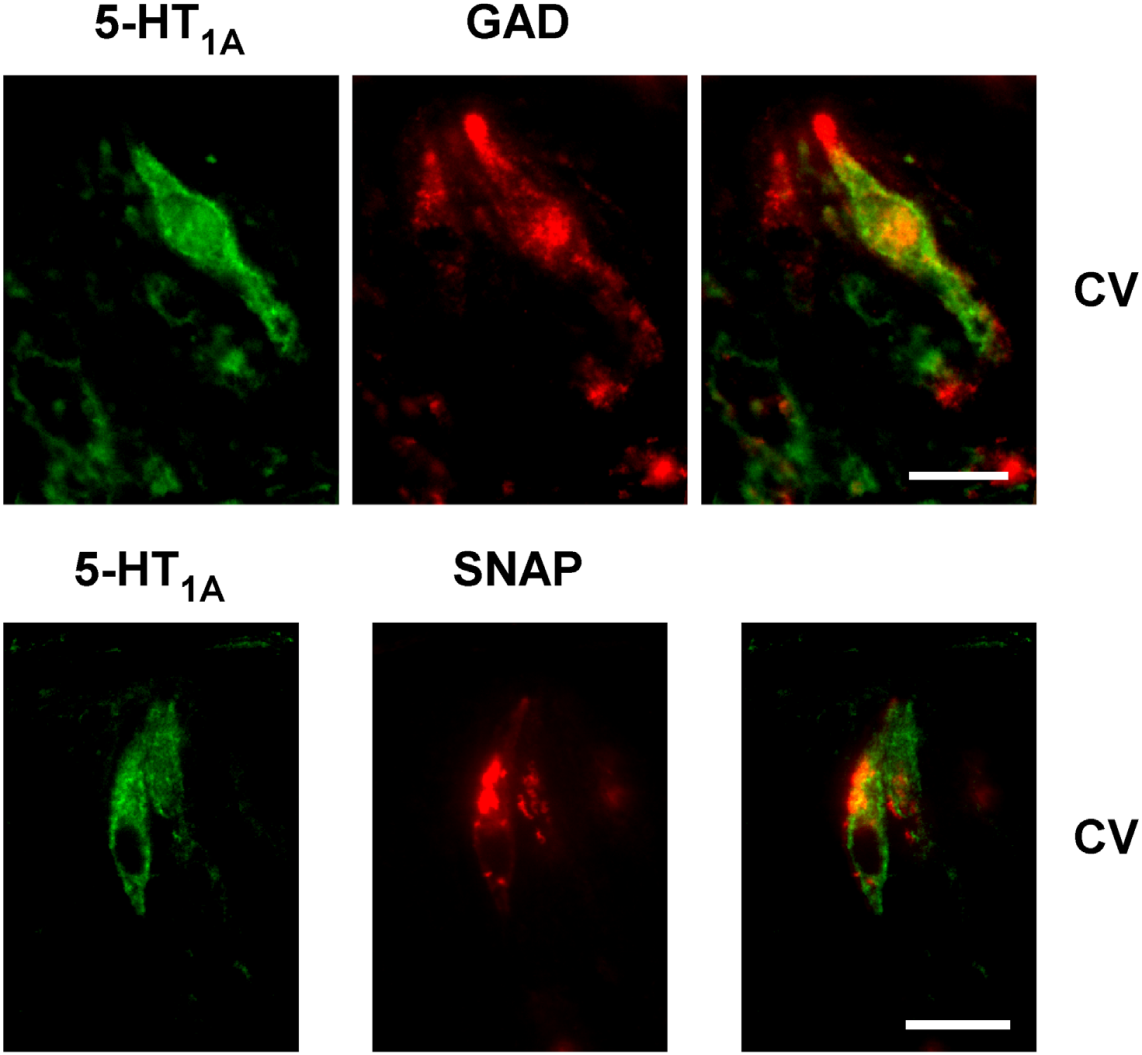
The co-expression pattern of 5-HT_1A_ was explored with two additional taste receptor cell markers, glutamic acid decarboxylase (GAD) and synaptosomal-associated protein 25 (SNAP25). Each is robustly expressed in type II cells of rat taste buds. Examples from circumvallate (CV) taste buds are illustrated. In each, strong overlapping patterns of immunopositive cells were observed. Scale bars are 15 microns.

Finally, a third set of phenotypic markers, which display less type II cell-specific expression in rat, were examined: synaptosomal-associated protein 25 (SNAP-25) and glutamic acid decarboxylase (GAD). In the rat, SNAP-25 is expressed predominantly in type II cells, with a minor degree of co-expression in type III cells [8]. The observed co-expression between 5-HT_1A_ and SNAP-25 resembled that of 5-HT_1A_ and gustducin (Figure 4; Table 3). In posterior taste buds, most of the 150 5-HT_1A_-immunopositive TRCs (89±1%; n = 134) co-expressed SNAP-25, which was expressed in 162 TRCs. Similarly, a large majority of the SNAP-25 cells (134 of 162 cells) co-expressed 5-HT_1A_. Results showed varying degree of overlap in the expression of 5-HT_1A_ with GAD (Figure 4). Only 34 of the 135 5-HT_1A_ expressing cells (18% ±2) co-expressed GAD, which was expressed in a total of 122 cells.

Co-expression patterns of the 5-HT_1A_ receptor with the tested phenotypic markers were statistically compared between posterior and anterior gustatory fields (posterior tongue vs. anterior tongue/NID respectively). Using the paired samples T-test, data revealed no significant differences between posterior and anterior fields for the co-expression of 5-HT_1A_ with gustducin (p = 0.062), NPY (p = 0.058), NPY-1R (p = 0.053), CCK-8 (p = 0.119), or GAD (p = 0.151). This suggests a similar pattern of co-expression for 5-HT_1A_ in posterior tongue and anterior tongue/NID (Table 3).

#### 5-HT1A expression and taste receptor molecules

Phenotypic characterization of the 5-HT_1A_ expressing TRC was continued with co-expression pattern with members of the T1R and T2R families of taste receptor molecules using single cell RT-PCR experiments on 51 TRCs cells isolated from the CV papillae of ten animals. The bitter receptor T2R9, the sweet/umami receptor T1R3, the sour receptor PKD2L1, the phenotypic markers gustducin and GAD were examined. Cytokeratin 8 (CK8), expressed in all mature TRCs but not in neighboring epithelial cells, was included as an internal positive control. All primers were optimized in separate RT-PCR experiments (n = 6) on cDNA reverse transcribed from total RNA extracted from individually excised whole CV papillae (see Table 2 for primer sequences). Negative control experiments were performed to ensure that PCR product did not arise from extraneous DNA. In one negative control, nuclease-free water was substituted for template in the RT-PCR experiment (water control). In another, ECF was collected from a region adjacent to a dissociated cell to test for extraneous DNA that might be present in the bathing medium (cell-free control). In other negative controls, primers were omitted. Gel analysis of PCR products from all negative control reactions showed no visible bands.

In the first group of experiments, ten single TRCs were randomly chosen from dissociated CV papillae and tested with three 5-HT_1A_ primers sets (5-HT_1A_[i], 5-HT_1A_[ii], 5-HT_1A_[iii]), each corresponding to a different region of the 5-HT_1A_ gene, along with primers for α-gustducin, T1R3, T2R9, and CK8. Four cells were negative for CK8 PCR product and were excluded. Of the remaining six cells, four showed PCR product of correct size for all three 5-HT_1A_ primers, confirming the specificity of these three primer sets. Three of the four cells expressing 5-HT_1A_ also expressed T2R9. The remaining cell expressed neither taste receptor molecule.

In subsequent experiments, one of the 5-HT_1A_ primers, 5-HT_1A_[iii], was chosen and phenotypic expression markers were expanded to include primer sets for CK8, gustducin, T1R3, T2R9, PKD2L1 and GAD on 35 individually isolated TRCs. Twenty-five cells were CK8-positive and included in data analysis. Combined data (Table 4) indicated that among all 51 single TRCs tested, 31 were CK8-positive, and thus were included in the data analysis. Of the CK8-positive TRCs, twelve showed specific bands for 5-HT_1A_. Of these twelve cells, ten expressed taste receptor molecules; four co-expressed T2R9; four co-expressed T1R3; two co-expressed T2R9 and T1R3; and two expressed neither taste receptor molecule. On the other hand, of the twelve T2R9-expressing TRCs observed, six did not express 5-HT_1A_, and of ten T1R3-expressing TRCs, four TRCs did not co-express 5-HT_1A_. Thus while almost all 5-HT_1A_ expressing TRCs expressed members of the TR families, the converse was not true. 5-HT_1A_-expressing TRCs may represent a unique subpopulation of T1R and T2R expressing TRCs.

**Table 4:** Gene expression in single taste cells.

Cell #	CK8	Gus	T1R3	T2R9	5HT_1A_(i)	5HT_1A_(ii)	5HT_1A_(iii)	PKD2L1	GAD
*1*	+	+	−	+	+	+	+		
*2*	+	−	+	−	−	−	−		
*3*	+	−	−	+	+	+	+		
*4*	+	+	−	+	−	−	−		
*5*	+	−	−	−	+	+	+		
*6*	+	+	−	+	+	+	+		
*7*	+	+	+	−			+	−	+
*8*	+	+	+	−			−	−	−
*9*	+	−	+	−			+	−	−
*10*	+	+	+	+			+	−	−
*11*	+	+	+	+			+	−	−
*12*	+	−	+	−			−	−	+
*13*	+	−	−	−			−	−	+
*14*	+	+	−	+			+	−	−
*15*	+	−	+	−			−	−	−
*16*	+	−	−	−			−	+	−
*17*	+	−	−	+			−	−	+
*18*	+	−	−	−			−	−	−
*19*	+	−	−	−			−	−	−
*20*	+	+	−	+			−	−	−
*21*	+	+	−	−			−	−	−
*22*	+	+	−	+			−	−	−
*23*	+	+	−	+			−	−	−
*24*	+	−	−	−			−	−	−
*25*	+	−	−	−			−	−	−
*26*	+	−	−	−			−	−	−
*27*	+	−	+	−			+	−	−
*28*	+	−	−	−			−	+	−
*29*	+	−	+	−			+	−	+
*30*	+	+	−	+			−	−	−
*31*	+	+	−	−			+	−	+

**Figure 5:**
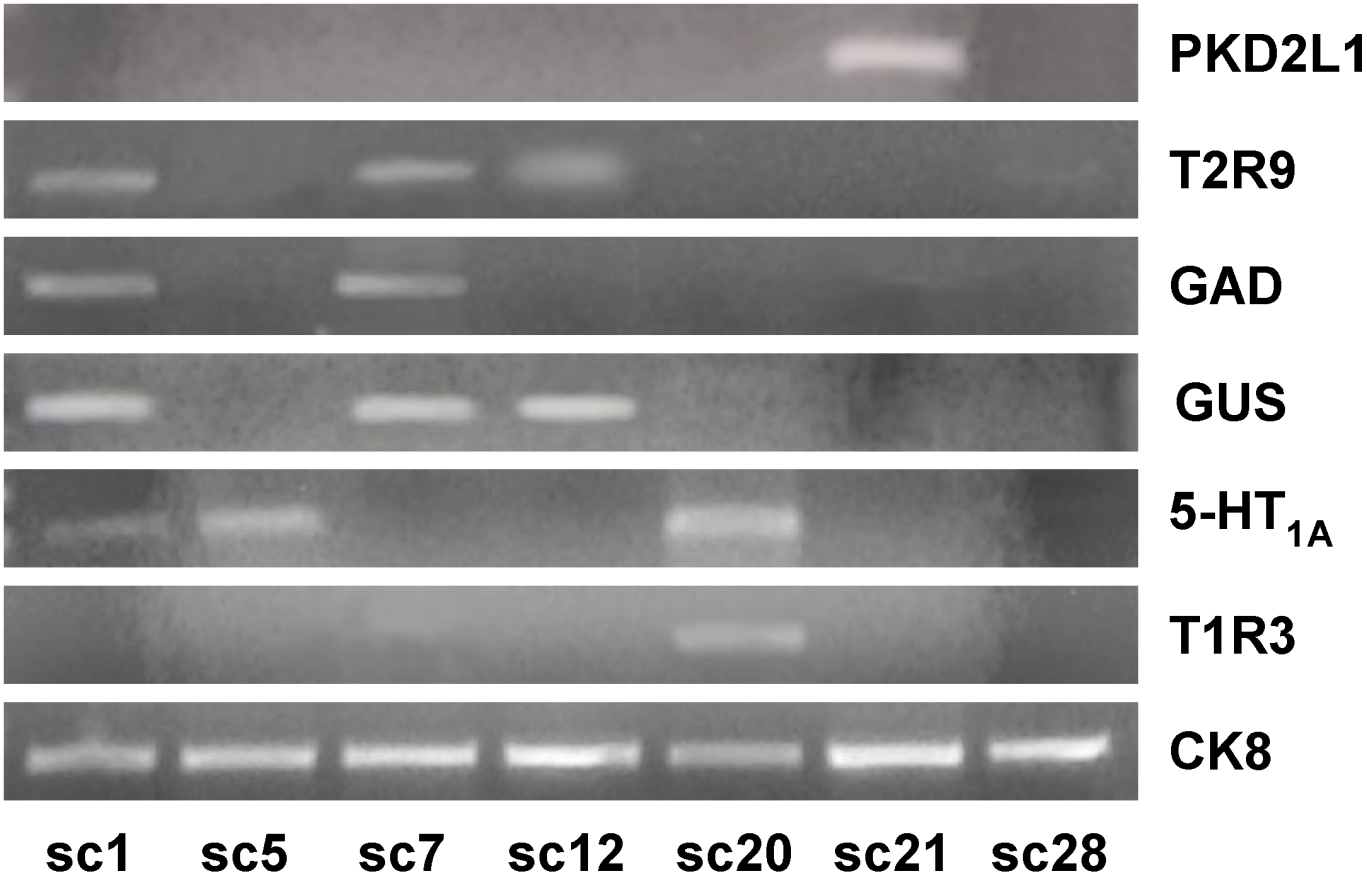
Gene expression patterns across individual taste receptor cells were examined using single-cell RT-PCR analysis. PCR products from seven individual taste receptor cells (sc1, sc5, sc7, sc12, sc20, sc21, and sc28) to seven separate PCR reactions are illustrated. The PCR reactions include positive control cytokeratin 8 (CK8), the 5-HT_1A_ receptor, the sweet/umami taste receptor molecule T1R3, the bitter receptor molecules T2R9, the sour taste receptor PKD2L1, and the phenotypic markers alpha-gustducin (GUS) and glutamic acid decarboxylase (GAD). For the most part, cells which expressed the 5-HT_1A_ receptor also co-expressed members of the T1R or T2R receptors. On the other hand, cells which expressed the sour receptor PKD2L1, which are type III cells, were not observed to express 5-HT_1A_. These data are in agreement with immunocytochemical data that 5-HT_1A_ expression occurs predominately in type II cells.

5-HT_1A_ co-expression patterns with single cell RT-PCR appropriately mirrored those observed with immunocytochemistry. For example, of the 12 5-HT_1A_ and 14 gustducin expressing TRCs, 7 co-expressed both proteins. Of the 6 GAD-positive cells observed, only two TRCs co-expressed 5-HT_1A_ and GAD. Of the two PKD2L1-positive cells observed in the 25 tested cells, none expressed either 5-HT_1A_ or other type II TRC-specific markers. As well, the expression pattern of multiple type II cell markers within a single TRC served as internal control. Of twelve TRCs that expressed T2R9, ten co-expressed its linked G-protein α-gustducin. Further, co-expression between gustducin and T1R3 was observed in five out of thirty-one TRCs. This pattern of co-expression agrees with previous studies that reported these two molecules co-localized in mouse TRCs [43], [47]. Of the six GAD-positive cells, three co-expressed T1R3, one co-expressed T2R9, and two co-expressed gustducin. These results agree with previous studies that report GAD co-expression with gustducin and T1R3 in mouse taste buds [8], [12], [56]. Overlap was not detected between the type III TRC marker PKD2L1 and any of the markers associated with type II cells, including 5-HT_1A_. Table 4 illustrates data from single cell RT-PCR experiments, and Figure 5 illustrates sample gel electrophoresis data from the RT-PCR products of seven different single TRCs.

On the other hand, unexpected co-expression patterns were also observed. Among the 32 CK8-positive TRCs, two co-expressed T1R2 and T2R9 (out of the total of the twelve T2R9- and ten T1R3-expressing cells). This was an unexpected outcome since the general consensus supports the notion that those two molecules are expressed in non-overlapping populations of TRCs [1], [28], [46], [52], [81]. While it is conceivable that genetic contamination may have occurred during the isolation process of single TRCs, recent studies have reported co-expression in T1R and T2R in a minor subpopulation of TRCs in zebrafish [58] and in the solitary chemoreceptor cells in mice [57]. Therefore, the co-localization of T2R and T1R in two cells in the current experiments may represent a small overlapping subpopulation T1R and T2R expressing TRCs.

### Chorda tympani neural recordings

To explore the physiological role of serotonin in the taste bud, chorda tympani (CT) neural responses were measured to lingually-applied tastants before and after intravenously injected serotonergic receptor antagonists. Control experiments were performed to determine vehicle effects and confirm the access of intravenously applied agents to taste buds.

#### Control saline injections

**Figure 6:**
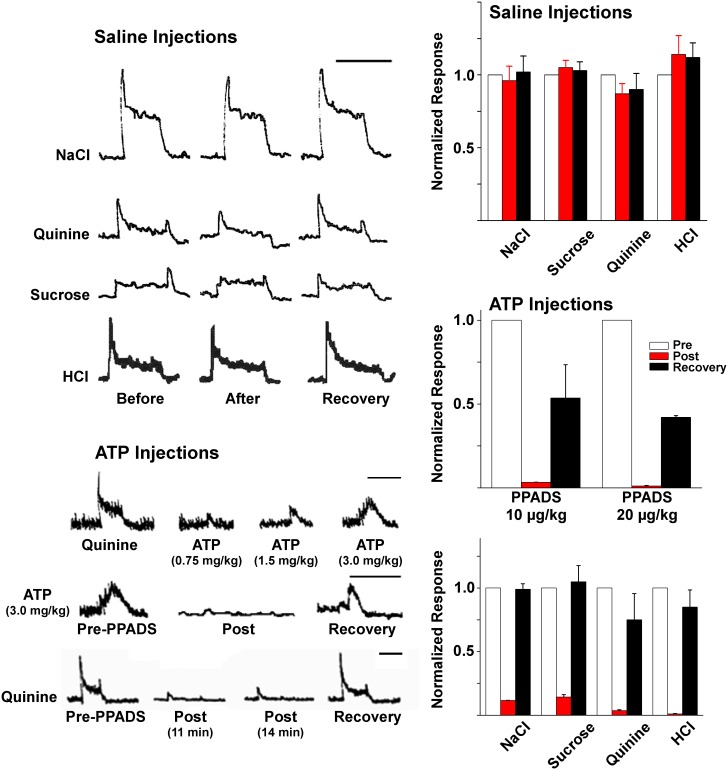
Intravenous injection of saline was without effect on chorda tympani nerve responses. Sample neural responses to 0.5 M NaCl, 0.03 M Quinine, 1.0 M sucrose, or 0.01 N HCl before and after a single bolus injection (300 µM) of saline are illustrated (upper left). Data are summarized in the histogram (upper right). On the other hand, intravenous injection of ATP produced transient neural responses which could be blocked by prior injection of the purinergic antagonist PPADS (lower left). Intravenously administered PPADS was also effective in blocking lingually applied tastant responses. Data summarizing the antagonistic effect of PPADS on ATP injection or tastant lingual stimulation are illustrated (histograms middle and lower right, respectively).

To examine vehicle effects on chorda tympani responses to taste stimuli, 300 µL single bolus jugular injection of saline was performed in eleven rats. Post-injection tastant responses were normalized to pre-injection response magnitudes recorded over the 20–30 minutes prior to injection. No change in baseline integrated neural activity was observed in any experiments during saline injection. Data averaged from all eleven experiments demonstrated no significant change of the neural response to any tested taste qualities when post-injection response amplitudes either immediately after (first three responses post injection) or up to one hour after saline injection (final 3 responses) were compared with pre-injection values (last 3 responses prior to injection; Figure 6). When pre-saline and post-saline mean response amplitudes were compared, the paired-samples T-test resulted in p-values greater than 0.05 (p = 0.085, 0.063, 0.191 and 0.193 for NaCl, sucrose, quinine and HCl, respectively).

#### Activation and antagonism of purinergic receptors

As an additional test that intravenously injected drugs could predictably affect gustatory neural responses, purinergic receptors were tested. As expected, injection of intravascular ATP produced neural excitations. ATP administered at three different concentrations (0.75, 1.5 or 3 mg ATP/kg BW, 300 µl) at 6 minute intervals, uniformly caused transient neural excitations (n = 3). Responses were bell-shaped and displayed dose-dependent magnitudes (Figure 6). Accompanying neural excitations, there was a transient dose-dependent increase in respiratory rate that lasted between 10 and 20 seconds produced by the transient ATP-induced increase in pulmonary artery pressure [2], [73].

The non-selective P_2_ purinergic receptor antagonist, PPADS, was highly effective in blocking neural responses produced by either intravascular injections of ATP or lingually applied tastants. Following injection of 10 mg/kg PPADS, mean CT response amplitude to 1.5 mg/kg ATP declined by 97% (n = 3; *p*<0.001). Responses recovered to half of their pre-injection amplitudes 50 minutes after injection. At 20 mg/kg, PPADS virtually abolished CT responses to ATP (98.8% inhibition, n = 3, *p* = 0.001). Responses recovered to almost half (42%) of pre-injection magnitude 62 minutes after PPADS injection. Additionally, 10 mg/kg PPADS (n = 3) resulted in significant reversible inhibitions of CT responses to all taste qualities (Figure 6). Immediately after injection, CT responses decreased 53% in mean amplitude to NaCl (*p* = 0.003), 82% to sucrose (*p* = 0.001), 79% to quinine (*p* = 0.001), and 75% to HCl (*p* = 0.001). PPADS at 20 mg/kg (n = 3) resulted in larger decreases in neural responses: (87% NaCl (*p* = 0.001), 85% sucrose (*p* = 0.002), 97% quinine (*p* = 0.001), and 97% HCl (*p* = 0.001)). Responses to all taste qualities displayed significant recovery within 45 minutes after 10 mg/kg PPADS injection and 60 minutes after 20 mg/kg injection. Collectively, these positive control experiments with ATP and PPADS provided confidence in the physiological preparation.

#### Effect of blocking 5-HT1A on CT responses to gustatory stimulation

Chorda tympani responses to lingual taste stimuli were recorded before and after single bolus injections of one of two tested 5-HT_1A_ receptor antagonists, WAY 100635 (10, 25, 100, or 200 µg/kg BW) or NAD-299 (175, 350 or 750 µg/kg BW).

**Figure 7:**
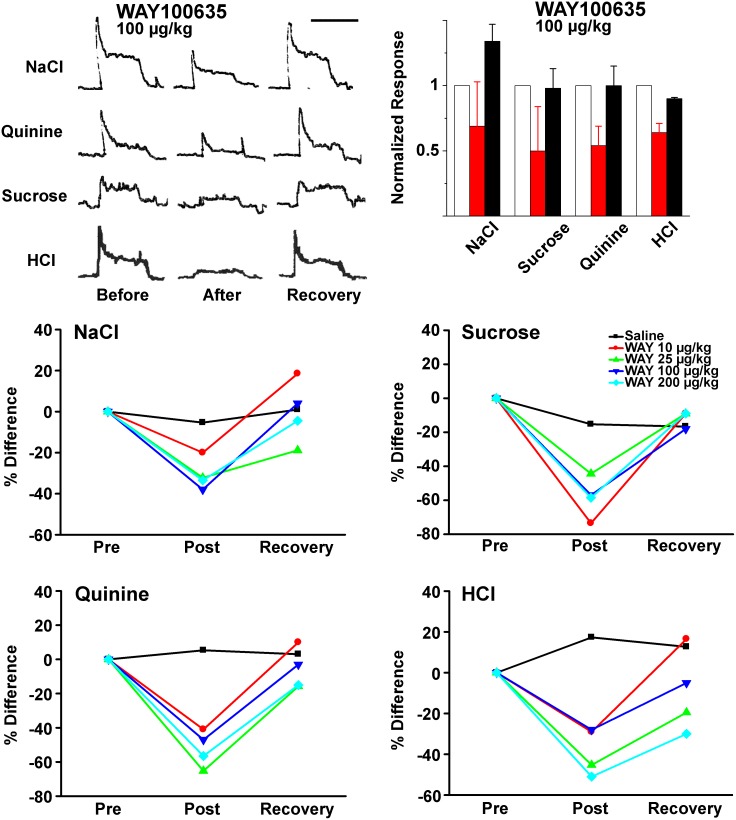
Intravenous injection of the 5-HT_1A_ antagonist, WAY100635, inhibits chorda tympani neural responses to lingually applied 0.5 M NaCl, 0.03 M Quinine, 1.0 M Sucrose, or 0.01 N HCl. Sample responses are illustrated prior to (PRE), immediately following (AFTER), and 45 to 60 minutes following (RECOVERY) a single bolus intravenous injection of 100 µM WAY100635 (upper left). Summarized data are presented in the histogram (upper right) for these four stimuli. In the lower figures, summarized data for four concentrations of WAY100635 are presented for each tastant and compared to vehicle (saline) injection.

Intravascular injections of WAY-100635 significantly reduced both phasic and tonic portions of the CT responses to all four tested taste stimuli. (Figure 7, upper left). Maximal drug effects were observed within the first ten minutes post-injection followed by a gradual recovery in response magnitude. On average, maximum recovery occurred 45 and 65 minutes post-injection. All tested concentrations of WAY-100635 inhibited neural responses. At 100 µg/kg (n = 8), CT responses were inhibited by 31% for NaCl (*p* = 0.009), 50% for sucrose (*p* = 0.025), 46% for quinine (*p* = 0.002), and 36% for HCl (*p* = 0.005; Figure 7, histogram, upper right). Each post-injection inhibition was significant compared to saline controls (*p*<0.05).

**Figure 8:**
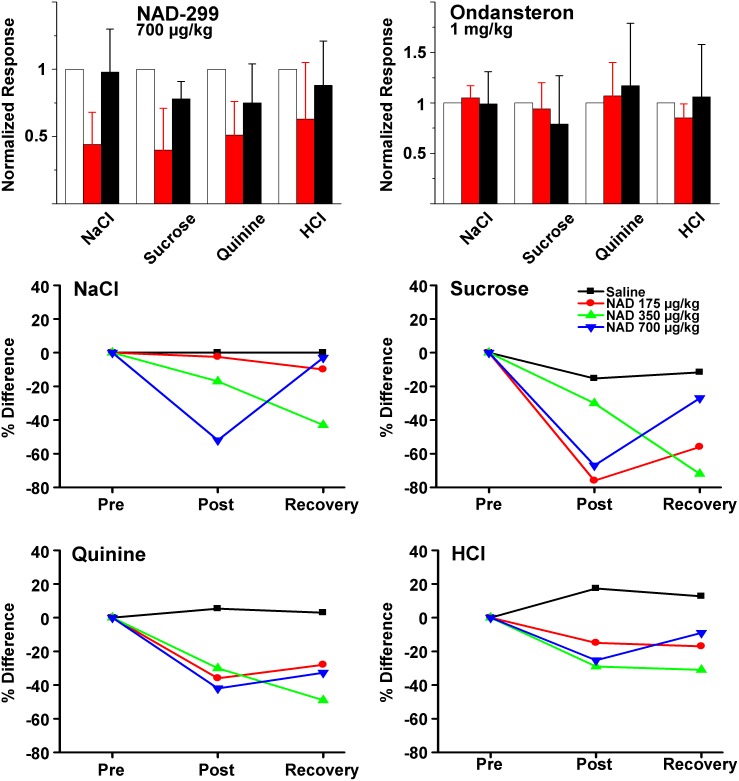
Another 5-HT_1A_ antagonist, NAD-299, similarly produced inhibitions of chorda tympani neural responses to applied lingual tastant solutions. Summarized data of 700 µg/kg NAD-299 are presented and concentration data for each tastant are presented to three concentrations of NAD-299 as well as vehicle injection. Unlike the 5-HT_1A_ antagonists tested, the 5-HT_3_ antagonist ondansetron was without noticeable effects on the magnitude of the chorda tympani neural responses (upper right).

To confirm the above findings, a second 5-HT_1A_ antagonist, NAD-299, was tested. Similar to WAY-100635, NAD-299 (175, 350, and 700 µg/kg) produced inhibition in neural responses to all gustatory stimuli (Figure 8, upper left, 700 µg/kg NAD-299). At each concentration, NAD-299 produced significant inhibitions of responses to all taste qualities with one exception (NaCl response amplitudes were reduced, but not significantly, post-NAD-299 175 µg/kg injection; Figure 8, lower graphs). Recovery from inhibition peaked between 40 and 65 minutes post-injection, similar to WAY-100635 treatment. Limited recovery was observed with NaCl responses following 175 µg/kg dosage, and NaCl, sucrose, and quinine responses following the 350 µg/kg dosage.

Injecting NAD-299 at 175 µg/kg (n = 5) significantly reduced the CT response to sucrose by 68% (*p* = 0.018), to quinine by 60% (*p* = 0.004), and to HCl by 37% (*p* = 0.008). At 350 µg/kg NAD-299 (n = 6), significant reductions were observed in the CT response to NaCl (27%; *p* = 0.014), sucrose (35%; *p* = 0.025), quinine (34%; *p* = 0.023), and to HCl (37%; *p* = 0.034). At 750 µg/kg NAD-299 (n = 6), inhibition was at 56% for NaCl (*p* = 0.001), 59% for sucrose (*p* = 0.001), 49% for quinine (*p* = 0.002), and 33% for HCl (*p* = 0.05).

Correlation between drug dose and the degree of inhibition was tested using Pearson’s correlation test. There was a statistically significant correlation between WAY-100635 dosage and neural inhibitions for sucrose (r = 0.987; *p* = 0.006), but not for NaCl (r = 0.803 *p* = 0.099), quinine (r = 0.536; *p* = 0.232), or HCl (r = 0.738; *p* = 0.131). Analysis of dose-effect relationship for NAD-299 revealed significant correlation between dosage and neural inhibitions for NaCl (r = 0.999; *p* = 0.015), but not for sucrose (r = −264; *p* = 0.415), quinine (r = −4.21; *p* = 0.362), or HCl (r = −0.866; *p* = 0.167). Although the amount of inhibition caused by either WAY-100635 or NAD-299 did not always statistically correlate to dosage, a clear pattern of significant inhibitions, followed by a recovery of neural responses, was observed after each administration.

These data suggest that 5-HT_1A_-mediated serotonergic activity is involved in the processing of sweet, sour, bitter and salt tastes and that, since antagonism of the 5-HT_1A_ receptor produced consistent neural inhibition to all taste qualities, the net effect of this serotonergic activation facilitates neural responses.

#### Effect of 5-HT3 antagonism on CT responses to gustatory stimulation

To examine a possible role for the 5-HT_3_ receptor, CT responses to gustatory stimuli were measured before and after single bolus injection of a 1000 µg/kg ondansetron, a 5-HT_3_ receptor antagonist. Data averaged from 10 separate experiments showed that no significant change in mean CT response amplitudes to any taste qualities up to 65 minutes post ondansetron injection. Paired-samples T-test for pre-injection and post-injection mean response amplitudes resulted in *p*-values greater than 0.05 (*p* = 0.295, 0.368, 0.396 and 0.081 for NaCl, sucrose, quinine and HCl respectively; Figure 8, top right). The only observed significant decline occurred to sucrose responses between 55 and 65 minutes post-injection. Given that this decline occurred long after drug injection, it is more likely it arose from other non-specific factors. Overall, no major modulatory role for the 5-HT_3_ receptor in neural processing of the tested gustatory stimuli was observed.

Taken together, findings from these nerve recording experiments demonstrate that: (1) systemically injected reagents are able to reach lingual tissue and target their corresponding receptors; (2) ATP is the main neurotransmitter of the taste bud and blocking its P2 receptors significantly reduces the bud’s neural output to the chorda tympani; (3) serotonin seems to act as a universal facilitator to all taste qualities; (4) this action of 5-HT appears to be mediated through its 5-HT_1A_ receptor subtype. In summary, serotonin’s 5-HT_1A_ receptors are expressed in a subset of type II TRCs that include sweet-, bitter-, and umami- processing cells. The physiological action of serotonin is to facilitate chorda tympani responses to sweet, bitter, salty, and sour taste stimuli through activation of 5-HT_1A_ receptors in type II TRCs.

## Discussion

Collectively, data in this study are the first to provide strong evidence for the functional role of the neurotransmitter serotonin in the peripheral gustatory system. We propose serotonergic transmission within the taste bud modulates taste signals at the TRC level during late transduction processes initiated by tastant stimulation. The expression pattern of serotonin and the 5-HT_1A_ receptor suggest a functional circuitry with serotonin release from type III cells acting on the 5-HT_1A_ receptor on a subset of type II cells to modulate ATP release preventing purinergic receptor desensitization on afferent fibers, thus enhancing the neural signal. In support of this hypothesis are anatomical data outlining a negative feedback circuitry between type III and type II cells and physiological data demonstrating functional evidence based on peripheral nerve responses. We suggest that activation of the 5-HT_1A_ receptor may regulate ATP release by modulating activity of the ATP-releasing channel, potentially through PIP_2_ resynthesis. We have observed, using patch clamp analysis, that serotonin slows the restoration of activity to potassium channels after adaptation by inhibiting PIP_2_ resynthesis [79]. PIP_2_ resynthesis is required to restore activity to many classes of ion channels, including connexion and pannexin channels. Hence, activation of the 5HT_1A_ receptor in TRCs may modulate ATP release by slowing the time course of the restoration of functional activity to ATP-release channels, thus reducing desensitization at purinergic receptors and in turn maintaining the gustatory neural signal to the brain.

In addition to the 5-HT_1A_ receptor, the taste bud expresses the ionotropic 5-HT_3_ receptor. At present, it is thought that this serotonergic receptor subtype is expressed on afferent gustatory nerve fiber terminals within the taste bud, rather than on TRCs [39]. The role of the 5-HT_3_ receptor in processing of peripheral gustatory information remains unknown and manipulation of the 5-HT_3_ receptor using an electrophysiological assay failed to produce a measurable functional modulation. While it had long been assumed that serotonin’s main action in the taste bud was as a neurotransmitter to the afferent nerve fiver, serotonin’s main action within the taste bud may be to act functionally as a gain modulator of the afferent neural output, acting primarily on TRCs within the bud via 5-HT_1A_ receptors rather than on gustatory afferent nerve fibers, the presumed location of 5-HT_3_ receptors in the taste bud.

### Phenotypic expression patterns of the 5-HT_1A_ receptor in taste buds suggest it acts on cells which release ATP

Although 5-HT_1A_ is known to be expressed in the taste buds of several species, its functional role remains uncertain. 5-HT_1A_ was first discovered pharmacologically in the rat taste bud [25] followed by immunocytochemical and mRNA expression data [39]. Functional studies have been limited to patch clamp analysis of 5-HT_1A_ expressing TRCs. In these studies, an inhibition of several ion currents have been reported including calcium-activated potassium current, sodium current, and calcium current [13], [25], [26]. Knowledge of 5-HT_1A_ activation at the single cell level only marginally predicts its function in peripheral gustatory physiology since the taste bud is a complex processing unit with multiple levels of cell-to-cell communication [24]. Knowing the cell type in which the 5-HT_1A_ receptor is expressed would be useful next step. Although serotonin-releasing cells are well known to be type III cells, and the 5-HT_1A_ expressing cell is known only to be paracrine to type III cells. Our earlier work suggested these cells to be type II cells [39]. Since type II cells release ATP [32], [40], [62], inhibition of these cells by serotonin would be functionally significant.

Our data provide strong evidence that the 5-HT_1A_ receptor is expressed in type II cells in the rat taste bud. General morphology, co-expression with multiple type II markers, and exclusion from a type III marker are all consistent with type II cell expression. Combined data from double-label ICC from anterior and posterior tongue, and single cell RT-PCR from posterior tongue, showed that the 5-HT_1A_ receptor is consistently co-expressed with phenotypic markers associated with type II cells (GUST, GAD, NPY, NPY-1R, CCK-8, SNAP, and GAD), including receptors for sweet and umami (T1R), and bitter (T2R) taste qualities. Further, immunocytochemical and single cell data showed that 5-HT_1A_ does not co-express with PKD2L1 and NCAM, phenotypic markers linked to type III cells. The association of NCAM with markers associated with type III cells has been well documented in rodent taste buds [69], [77]. As well, PKD2L1, a member of the transient receptor potential channel (TRP) family and candidate sour taste receptor, is expressed exclusively in a subset of type III cells [30]. Finally, without testing a type I marker, expression of the 5-HT_1A_ receptor in the type I cell remains an open question. However, expression in type I cells may be unlikely. The morphology of 5-HT_1A_ immunopositive cells, while not a definitive criteria by itself, is inconsistent with type I cell expression. Characteristically, 5-HT_1A_ immunopositive cells display large round soma which is characteristic of type II cells. At present type I expression cannot be excluded.

Collectively, 5-HT_1A_ phenotyping experiments suggest that this receptor is not expressed in all type II cells. For example, 5-HT_1A_ was not expressed in all gustducin-positive cells or in all T2R-positive cells. Gustducin is thought to be expressed in all cells that process bitter taste [29], and all gustducin-positive cells have the ability to release ATP [53]. Therefore, the incomplete pattern of co-expression between gustducin/T2R and 5-HT_1A_ observed in the present study suggests that not all bitter-transducing cells are directly targeted by serotonergic modulation. Hence, subsets of bitter-transducing TRCs may contribute differentially to the neural response; *i.e.*, not all TRCs which express bitter transduction machinery may equally activate the afferent nerve. Bitter-sensitive TRCs may functionally differentiate based on a number of parameters such as cell age, subset of T2R molecules expressed, or proximity to an afferent terminal. Additionally, TRCs may only express a full repertoire of required molecular transduction units at a defined window during cell lifespan. Whether all TRCs expressing T2R molecules contribute equally or differentially to afferent nerve activation is, at present, an open question. The possibility that serotoninergic modulation operates on only a cohort of bitter receptor cells which in turn contribute differentially to the neural response remains an interesting, though unproven, hypothesis. If true, the subset of T2R-expressing TRCs that additionally express 5-HT_1A_ would represent a functionally distinct group of cells in the bud capable of strong influence over the afferent neural response.

Along this line, the co-expression pattern of the 5-HT_1A_ receptor with neuropeptides and neuropeptide receptors, all known to be expressed in type II cells, suggests that serotonin may participate in the complex network of neuropeptide signaling within the taste bud. Within the taste bud, cholecystokinin, the CCK-1 receptor, NPY, and vasoactive intestinal polypeptide are predominantly co-expressed in a subset of type II cells which are paracrine to another subset of type II cells expressing the NPY-1 receptor. CCK and NPY, which are co-expressed, act in an autocrine stimulatory manner and a paracrine inhibitory manner, respectively [80]. In the present study, immunocytochemical results revealed that 73% of the TRCs expressing NPY and 66% of those expressing NPY-1R co-express 5-HT_1A_, a pattern consistent throughout taste buds in posterior tongue, anterior tongue, and the nasoincisor ducts. These findings imply that in late transduction mechanisms, 5-HT_1A_-expressing cells themselves may be subject to complex modulation, such as by the neuropeptide signaling system, and hence there may be multiple layers of modulation influencing ATP release within the taste bud.

### A functional role for serotonin in the taste bud

Electrophysiological data demonstrate that chorda tympani neural responses to tastant stimuli are significantly diminished by administration of a 5-HT_1A_ antagonist. Responses were reduced in magnitude with little effect on temporal properties, i.e. phasic and tonic responses. These observations are consistent with reduced neurotransmitter release from the TRC without alteration of other mechanisms, such as adaptation. Positive control experiments demonstrated that intravenous agents reach the taste bud as evidenced by injection of ATP and by strong inhibition of all taste responses to the ATP antagonist PPADS. Inhibition was observed with two 5-HT_1A_ antagonists and was not replicated by either vehicle injection or by a 5-HT_3_ antagonist. Further, our data are in lines with those who have previously used this technique [68]. We believe that the combination of three positive control experiments, one negative control experiments, and the repeatability of two 5-HT_1A_ have produced a strong and reliable electrophysiological data set.

In total, these observations are consistent with the notion that the 5-HT_1A_ receptor plays a facilitating role in taste transduction mechanisms of multiple taste qualities. Because serotonin’s action at the cellular level is inhibitory, serotonin acts during transduction by a process similar to disinhibition. We suggest the site of this disinhibition is regulation of ATP release preventing purinergic receptors desensitization. As a neurotransmitter in the taste bud, ATP activates P_2_X_2_/P_2_X_3_ purinoceptors on afferent nerve endings to transmit gustatory signals [16]. P_2_X_3_ channels display high rates of desensitization [11], [21], [61]. By regulating ATP release through its release channels, such desensitization would be prevented, thus facilitating the neural response. Additionally ATP has neuromodulatory roles within the taste bud including both positive autocrine and paracrine feedback mechanisms [15], [31]. ATP may activate P_2_Y_1_ and P_2_X_2_ receptors on type II cells [20], [38] via autocrine routes, thereby auto-enhancing and triggering its own release respectively. ATP may also activate P_2_Y receptors expressed on type III cells in a paracrine manner, including serotonergic cells, ultimately triggering serotonin release [31].

Serotonin can also be directly released from type III cells upon their stimulation with sour stimuli [33]. Serotonin is released from TRCs in response to multiple tastant qualities in mouse taste buds [33], which agrees with our electrophysiological results of serotonin’s influence on multiple taste qualities. Subsequently, serotonin activates 5-HT_1A_ receptors expressed in a group of type II cells through a negative paracrine feedback, inhibiting those cells and the release of ATP. Supporting this notion is the observation that in mice taste-evoked ATP release from type II cells was inhibited by serotonin and enhanced by blocking 5-HT_1A_ with WAY-100635 [31]. Recent quantitative studies of rodent taste buds have documented contain that about 14% of taste receptor cells in a bud are serotonergic; hence, a typical bud of 50 to 100 cells would contain an average of 7 to 13 cells serotonin cells [45]. Hence, it is easy to envision that serotonin could exert significant and measurable modulation of the neural response, particularly with the close apposition of type II cells with a single type III cell. Therefore, blocking the 5-HT_1A_-receptor-mediated serotonergic transmission in the taste bud would be logically assumed to disinhibit ATP release, and thus enhance the stimulus for synaptic P_2_X purinoceptors.

The identity of the ATP release channel is not known with certainty. Purinergic neurotransmission in the taste bud does not require traditional synapses, as found on type III cells [32], [62], [63]. Instead release is nonvesicular through channels whose candidates include connexins, pannexin-1, and a newly discovered release channel, CALHM1 [32], [62], [72]. As CALHM1 knockout mice do not have impaired responses for all taste qualities, multiple ATP-release mechanisms may exist. Whether activation of the 5HT_1A_ receptor targets some or all of these channels is not known. Several studies have shown that ion channels [9], [36], [44], [64], [70], [71] including connexin and pannexin hemichannels [27], [70] are regulated by PIP_2_ levels. We are unaware if the sensitivity of CALHM1 to phospholipids has yet been tested. Data from preliminary patch-clamping work have also demonstrated that pharmacologically altering PIP_2_ re-synthesis modulates the excitability of TRCs [79]. The cellular inhibition caused by the activation of 5-HT_1A_ receptors reduces PIP_2_ resynthesis [51], [55], [59] and, consequentially, would inhibit the activity of connexin/pannexin channels, resulting in a reduction in ATP release.

Finally, if the 5HT_1A_ receptor is involved in modulating the peripheral neural response to taste stimuli, one might predict that alterations of the serotonergic system might result in gustatory perceptual changes. For example, in humans, circumstances in which serotonin is altered, such as depression, are associated with taste disturbances. Along this line, there are a few relevant examples of experiments in human and rodent models with the selective serotonin reuptake inhibitor (SSRI) paroxetine. Taste thresholds to sweet and bitter in healthy humans with paroxetine were reduced [22]. These observations are consistent with increased serotonin concentration in the taste bud which might result in greater taste acuity reflected as either lower thresholds and/or a shift in perceptual intensity. In rodents, behavioral experiments with paroxetine in brief access test and operant condition paradigms have been conducted. In a brief access taste test, paroxetine did not affect concentration-dependent licking to taste stimuli but produce a decrease in the number of trials that water-deprived rats initiated when quinine was the stimulus and that non-deprived rats initiated when sucrose was the stimulus [49]. Systemically increased serotonin did not affect consummatory behavior but, at least in some conditions, did attenuate appetitive behavior. The effects observed in the present paper would agree with the lack of effect on consummatory behavior since 5-HT_1A_ receptor activation influences modulation rather than detection. In an operant conditioning task, water-deprived rats had lower breakpoints for water and quinine when injected with paroxetine compared with vehicle [48]. The authors conclude that a general systemic increase in serotonergic activity decreases the reward value of both preferred and avoided fluids under different motivational states and suggest these findings to be consistent with those from the brief access taste test. For the most part, these studies are complicated by the inability to delineate a primary site of action of serotonin since such treatments affect both central and peripheral serotonergic systems. Nevertheless, the results display an internal consistency across animal and human models that altering serotonin levels in the taste bud may influence peripheral taste detection and that this alteration may correlate higher serotonin levels with overall higher taste acuity.

In summary, the present study strongly supports the notion that serotonin is a crucial player in a finely tuned feedback mechanism involving 5-HT_1A_ receptors, ATP, and the P_2_X_2_/P_2_X_3_ purinoceptors. Data suggest that serotonin’s primary role in the mammalian taste bud is to modulate taste signals prior to the final neural output and this modulation likely occurs by controlling the release of the gustatory neurotransmitter ATP from 5HT_1A_-expressing type II cells. Serotonin thus plays a role in the complex set of late gustatory transduction mechanisms that take place at the cellular level within the taste bud during the processing of taste information.
